# The Iron Curtain: Macrophages at the Interface of Systemic and Microenvironmental Iron Metabolism and Immune Response in Cancer

**DOI:** 10.3389/fimmu.2021.614294

**Published:** 2021-04-27

**Authors:** Angela DeRosa, Avigdor Leftin

**Affiliations:** ^1^ Department of Pharmacological Sciences, Stony Brook University School of Medicine, Stony Brook, NY, United States; ^2^ Department of Radiology, Stony Brook University School of Medicine, Stony Brook, NY, United States

**Keywords:** cancer systems, immunotherapy, iron metabolism, macrophage polarization, tumor microenvironment

## Abstract

Macrophages fulfill central functions in systemic iron metabolism and immune response. Infiltration and polarization of macrophages in the tumor microenvironment is associated with differential cancer prognosis. Distinct metabolic iron and immune phenotypes in tumor associated macrophages have been observed in most cancers. While this prompts the hypothesis that macroenvironmental manifestations of dysfunctional iron metabolism have direct associations with microenvironmental tumor immune response, these functional connections are still emerging. We review our current understanding of the role of macrophages in systemic and microenvironmental immune response and iron metabolism and discuss these functions in the context of cancer and immunometabolic precision therapy approaches. Accumulation of tumor associated macrophages with distinct iron pathologies at the invasive tumor front suggests an “Iron Curtain” presenting as an innate functional interface between systemic and microenvironmental iron metabolism and immune response that can be harnessed therapeutically to further our goal of treating and eliminating cancer.

## Introduction

Defining patient prognosis, potential precision therapeutic avenues, and ultimately survival outcomes on the basis of metabolism is complicated by the need to integrate macroenvironmental and microenvironmental processes and multi-cellular metabolic systems interactions. Systemic metabolism establishes a unique profile of metabolites in the tumor microenvironment (TME), but distribution of these metabolites in the TME and their characterization is complicated by the multi-cellular dynamic composition of the tumor that introduces spatial heterogeneity of the metabolite distribution due to inter-cellular competition for metabolites that can promote tumor growth and hinder effective anti-tumor responses (1–5). This intersection of metabolism and cellular function in the TME is increasingly recognized as being of critical importance in cancer immune response. Metabolic gradients in the TME and systemic changes in metabolism alter immune cell activity and notably plays a prominent role in mediating immunotherapeutic responses (6, 7).

Of the various cells involved in cancer, macrophages play a central role in systemic and microenvironmental metabolism that has prominent effects on immune response in cancer. Tumor associated macrophages (TAMs) are implicated in all stages of cancer from tumorigenesis, to metastasis outgrowth, and therapy response as they plastically change their immune response according to local and systemic cues (8, 9). Metabolically, in non-malignant diseases and homeostatic contexts, macrophages exhibit a unique metabolic trait throughout the body in diverse tissue microenvironments in that macrophages can shift the fate of immune response in a manner dependent upon their central function in iron recycling (10–12). Investigators are increasingly focused on similar connections between cancer, TAM immune response, and iron metabolism. Thus, here we review our current understanding of macrophages in metabolic iron handling and immunologic response in cancer. To contextualize the role of TAMs in iron handling we review macrophage’s dual roles in iron handling and immune response both systemically in organs throughout the body, and in various tumors. Further, we detail how macrophages are central to the axis of immune system and iron metabolism in cancer therapy and demonstrate how harnessing either their iron level or immune response jointly effects the other enhancing our ability to treat cancer. These new insights will support new opportunities for therapeutic interventions at the multi-systems level.

## Regulation of Iron Metabolism By Macrophages

### Molecular Mechanisms of Iron Handling

Macrophages are involved in controlling iron import, export, and storage. These functions are regulated post-transcriptionally. mRNA-binding iron regulatory proteins 1 and 2 (IRP1 and IRP2) mediate cellular iron uptake, transport, storage and utilization in macrophages and hepatocytes (13). IRPs bind their target transcripts to regulate protein transcription and iron regulatory elements in response to iron levels in the body (14). In iron deficient conditions, IRPs will bind with high affinity to the iron regulatory element (IRE) in heavy and light chain ferritin mRNA and inhibit their translation to prevent storage of iron. In replete iron cells, IRP binding to IRE is reduced to allow for degradation of TfR1 mRNA and translation of ferritin mRNA to support cellular iron storage (15).

Macrophages take-up different forms of iron. Transferrin bound, and non-transferrin bound free iron (NTBI) enter the macrophage *via* specific cell surface receptors (16). The transferrin receptor (TfR) sits on the macrophage cell surface and recognizes transferrin-bound iron, or holo-transferrin, which becomes endocytosed upon binding (16–18). Similarly, receptors for lactoferrin (LFN), a member of the transferrin family that binds iron and has numerous functions (19), are present on many immune cells, including macrophages (20). NTBI can be transported by ZIP14, a ZIP family member of metal ion transporters, where it is upregulated on human primary macrophages under inflammatory conditions (21). NTBI can also be taken up by the divalent metal transporter 1 (DMT-1), also known as SLC11A2 which is associated with duodenal cytochrome B on the surface of the macrophage. In the endosome at low pH, iron is released if bound to transferrin, then reduced and stabilized by the endosomal reductase six-transmembrane epithelial antigen of the prostate 3 (STEAP3), from ferric iron (Fe^3+^) to ferrous iron (Fe^2+^) and transported through DMT-1 into the cytosol for ferritin storage, metabolic cofactor processes, or export through ferroportin (FPN), the cellular iron exporter (16).

Macrophages recycle heme iron from phagocytosis of senescent erythrocytes. Senescent red blood cells (RBCs) present cell surface markers to be recognized by macrophage scavenger receptors for phagocytosis. CD91 and CD163 are two scavenger receptors expressed at high levels on the surface of the macrophage. CD91 binds hemopexin-bound heme iron while CD163 bind both free iron and haptoglobin-hemoglobin complexed iron (22). RBCs are engulfed and digested by macrophages *via* erythrophagocytosis. RBCs phagocytized by the macrophage will be degraded, iron will be released from heme in the phagolysosome by heme oxygenase 1 (HO-1), transported to the cytosol of the macrophage, and processed for storage or recycling (11). NTBI can be exported through FPN out of the cell, supported by the ferroxidase ceruloplasmin, and loaded into transferrin to be transported to other target cells (16).

Macrophages store iron in ferritin. Ferritin is normally found within cells but can also be found in plasma. Ferritin is an iron storage protein complex consisting of 24 molecules of light (FTL) and/or heavy (FTH) chains. Extracellular ferritin can bind cell surface ferritin receptors, mainly including heavy chain H-ferritin receptor T cell immunoglobin and mucin domain-2 and light chain L-ferritin receptor scavenger receptor member 5, mediate the uptake of ferritin-bound iron (16). After uptake, NTBI is freed from ferritin protein and processed further by the cell for export.

### Macrophages in Systemic Iron Metabolism

**Figure 1:**
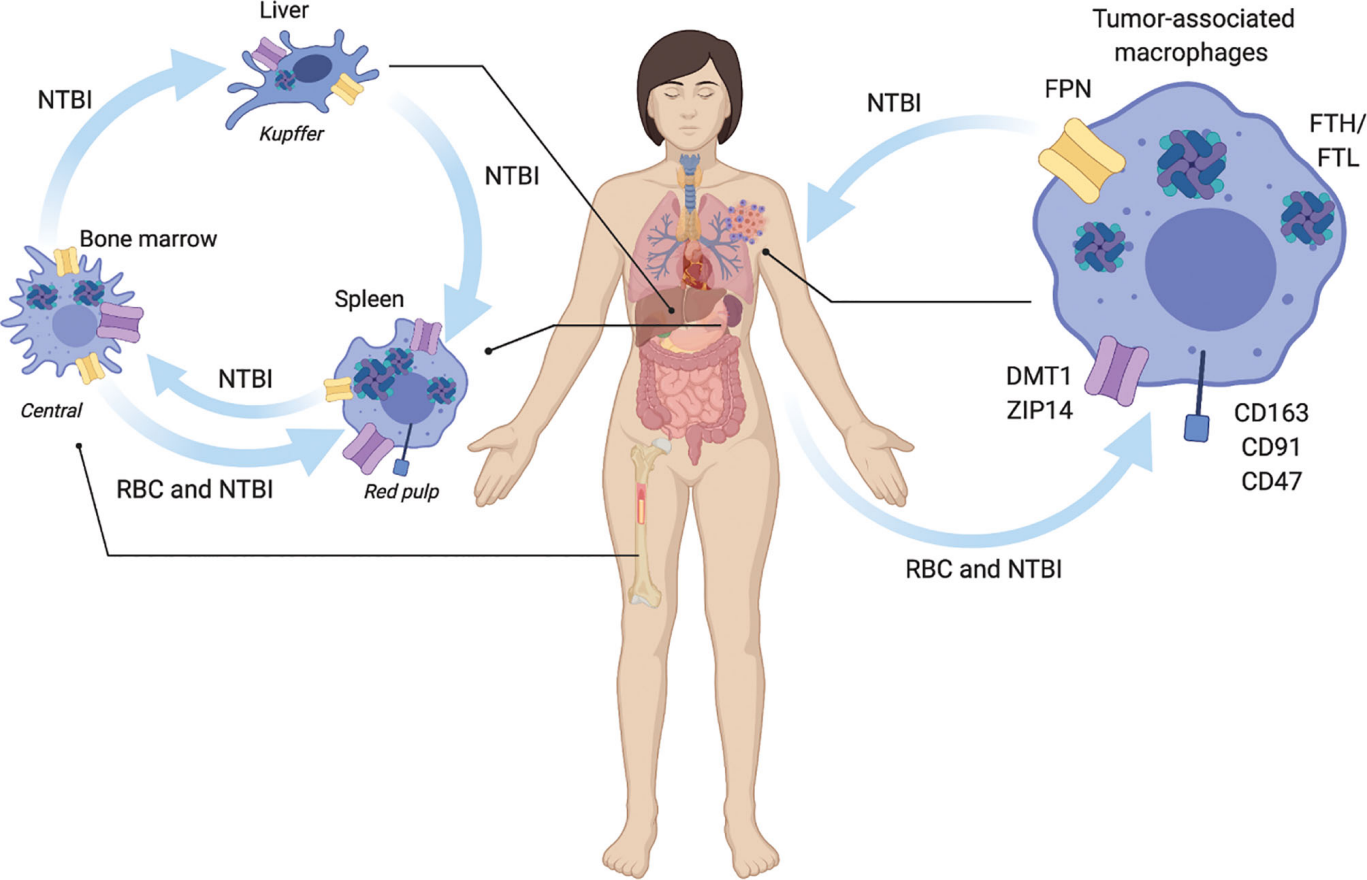
Macrophage regulation of systemic iron metabolism. Macrophages are central regulators of iron metabolism systemically throughout the body where they regulate largely unidirectional flux of non-transferrin bound iron NTBI, and red blood cell (RBC) erythrophagocytosis. Macrophages across the body and in tumors share similar uptake, storage and release mechanisms. NTBI iron is taken up by ZIP14, and DMT1. RBC are recognized for phagocytosis *via* CD163, CD91, and CD47 receptors. NTBI is stored as ferritin heavy chain (FTH) and light chain (FTL) complexes. NTBI is released by ferroportin (FPN). In liver Kupffer cells integrate inflammatory cues with NTBI iron recycling to regulate iron homeostasis. Spleen red-pulp macrophages respond to systemic metabolic iron needs by phagocytosis of senescent red blood cells (RBC) and export of recycled NTBI. Bone marrow central macrophages process NTBI received from the periphery to support heme synthesis during erythropoiesis. In the tumor, tumor associated macrophages similarly recycle NTBI and RBC.

Iron is regulated systemically by specific organs including the liver, spleen, and bone marrow. Tissue-specific macrophage populations are present within these organs as distinct phenotypic subsets (23). These resident macrophages have broad roles in removing debris, such as senescent and apoptotic cells, they help in development by promoting angiogenesis and bone break-down, and in regulation of metabolism (23). They also aid in controlling iron homeostasis at the local and systemic levels (24). Here we review the molecular mechanisms by which macrophages regulate iron, and present examples of macrophages in systemic contexts where they perform these iron recycling roles to integrate our forthcoming observations of TAM iron handling and immune response within the larger context of macrophage iron recycling systems of the body such as drawn schamtically in **Figure 1**.

The liver is a major center for iron regulation. Hepatic hepcidin production regulates systemic iron export by promoting the internalization of the iron exporter FPN on macrophages and other cells, lowering circulating NTBI concentrations (24). This leads to a decrease in the concentration of systemic iron and the accumulation of iron within iron-handling macrophages such as Kupffer cells in the liver. Liver Kupffer cells take up and store NTBI from senescent erythrocytes and release it in response to systemic need. In instances of chronic inflammation over-accumulation of liver iron leads to less iron circulation throughout the body and decreased RBCs production (25, 26) in anemia of chronic disease (27, 28).

The red-pulp macrophages of the spleen filter the blood of senescent erythrocytes acting as a quality control mechanism to regulate circulating RBCs and plays important roles during inflammation by serving as a depot of immune cells. Splenic macrophages rapidly clear senescent RBCs from the blood that do not express the CD47 “don’t eat me” cell surface signaling molecule and recycle the heme iron they contain. The extracted NTBI is trafficked within red pulp macrophages to be stored in ferritin, utilized by the mitochondria, or exported to other organs, such as the bone marrow.

In the bone marrow, erythroid island macrophages express high levels of iron regulation protein machinery including TfR, HO-1, and FPN to support RBC heme production (29). Osteoclasts are viewed as bone marrow resident macrophages, but non-osteoclast macrophages exist as well (30). Due to their bone repair function, non-osteoclast resident bone macrophages can be examined to determine their specific function as iron-handling regulatory cells. Iron release was shown to be necessary for osteoclastogenesis and general skeletal homeostasis and a population of resident bone marrow macrophages in mice. Thus, iron metabolism is implicated in osteoclasts, bone macrophages that drive bone reabsorption and bone healing.

Lastly, most cells and tissues participate in regulation of iron metabolism, as iron is critical for their function and is potentially harmful if accumulation or depletion is left unchecked. For example, systemic iron metabolism is tightly regulated by the kidney to properly carry out cellular functions such as erythropoiesis *via* erythropoietin, hypoxia signaling, mitochondrial respiration and DNA synthesis, while avoiding toxicity from free iron (31). Oxidative metabolism associated with renal iron overload is associated with renal cell carcinoma development (32). Ferroptosis, an iron-dependent form of cell death is also identified and associated with renal ischemia-reperfusion injury (33). Cells and tissue with high mitochondrial respiration needs, such as skeletal muscle myocytes, require iron for respiration and myoglobin production. This is due to the role of iron as cofactor for many of the respiratory chain proteins. Macrophages present in muscle express higher levels of haptoglobin, HO-1, CD163, and ferritin, suggesting they sequester myoglobin iron when released from damaged monocytes in response to acute injury and dysregulation of iron homeostasis in muscle can lead to myopathies under iron deficiency or aberrant oxidative stress which contributes to muscular atrophy. After injury, skeletal muscle macrophages upregulate FPN to release iron and contribute to myofiber regeneration, indicating that iron is necessary for muscle healing (24).

## Macrophage Polarization and Iron Metabolism

As we have shown above, macrophages play a central role in systemic iron metabolism. Cells of the innate immune system more famously play essential roles in inflammation and systemic host defense (34, 35). In response to local damage, detection of pathogens, or stimulation with lipopolysaccharide in the laboratory, macrophages become activated and polarize towards an “M1” like phenotype (35). These classically activated, pro-inflammatory M1 macrophages are characterized by high levels of pro-inflammatory cytokines, such as interferon-γ (IFN-γ), tumor necrosis factor alpha (TNF-α), interleukin 1 (IL-1), interleukin 6 (IL-6), produce inducible nitric oxide synthase (iNOS) and high reactive oxygen species (ROS) to promote bactericidal and anti-tumor activity (36). Stimulation with a variety of other cytokines and signaling molecules, including IL-4, IL-10, IL-21, and transforming growth factor – β (TGF-β) triggers a shift in macrophage polarization to an “M2” like phenotype (37). This subset of alternatively activated macrophages function in response to tissue damage and aid in repair, matrix remodeling, angiogenesis, and tumor promotion (38). M2 macrophages contribute to inflammation resolution by initiating wound repair. They produce angiogenesis mediators such as TGF-β, vascular endothelial growth factor (VEGF), and epidermal growth factor (EGF) (39).

The iron handling function of macrophages is coupled with shifts in their polarization over the course of their immune response. Macrophages are found polarized in the M1 state and engaged in iron sequestration as part of the acute inflammatory signaling response to bacterial and fungal infection (40, 41). The M1 iron retaining macrophages store ferritin iron and reduce import and export to prevent pathogens and non-self-cells from utilizing iron to proliferate (16). Along with inflammation markers, M1 macrophages are characterized by low expression levels FPN, CD163, and HO-1 while expressing high levels of ferritin to support this iron retention phenotype. With respect to iron metabolism, M2 macrophages demonstrate more of an iron release phenotype at sub-acute stages of immune resolution. Unlike M1, M2 macrophages have high expression levels of FPN, CD163, HO-1, and low expression levels of ferritin, contributing to an iron donating phenotype.

## Iron Metabolism and Macrophages in Cancer

**Figure 2:**
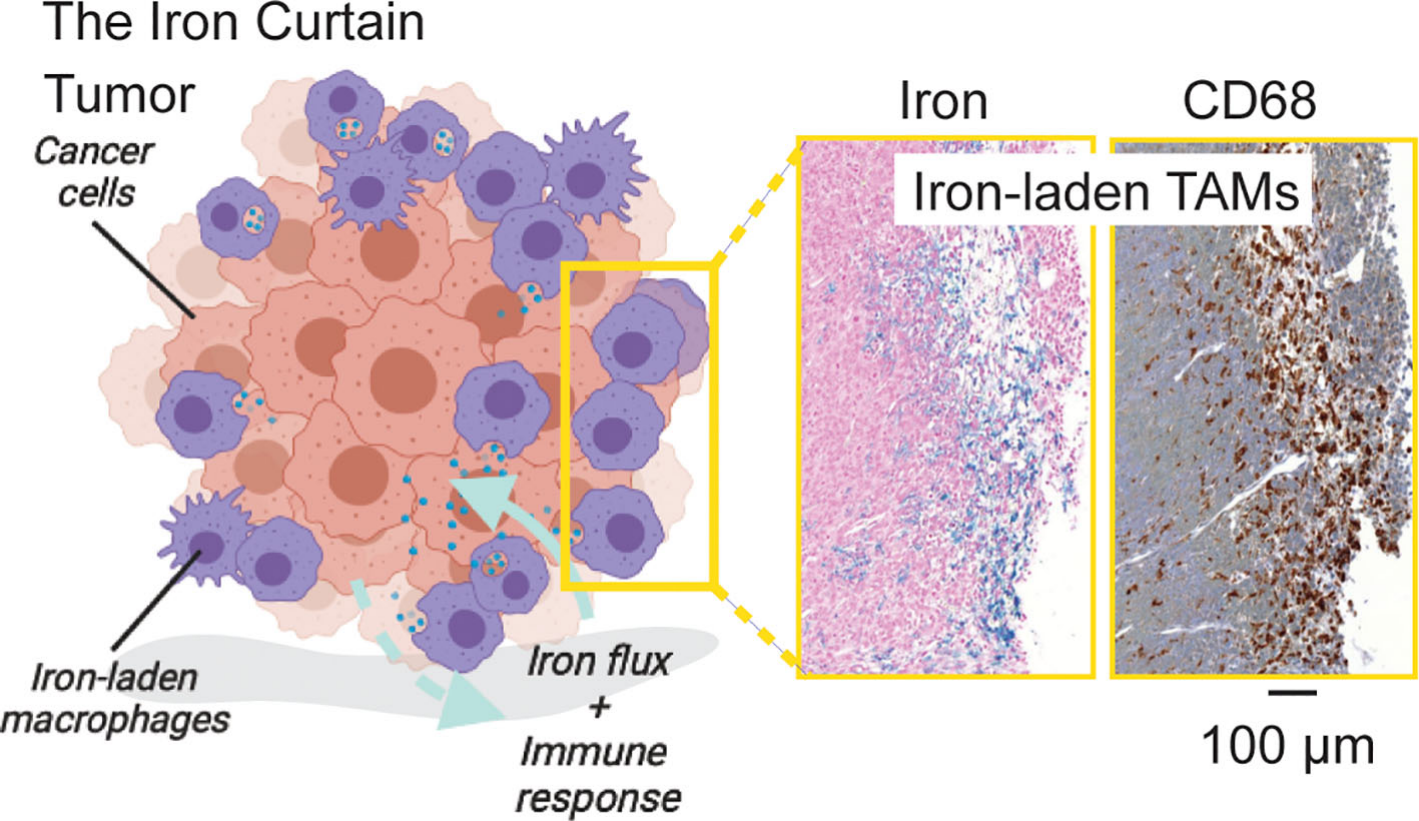
The Iron Curtain. Iron-laden macrophages occupy a unique cellular niche in the tumor where they act as an interfacial boundary mediating systemic and microenvironment metabolic flux and immune response. Prussian Blue iron histochemistry which is specific for ferric iron deposits from endogenous hemosiderin (shown here) or iron nanoparticle contrast agent (not shown) in such macrophages beside pan-macrophage CD68 immunohistochemical staining reveals a distinct spatial pathology of such iron-laden macrophages suggestive of an **“**Iron Curtain**”** where colonies of TAMs exhibiting similar iron accumulation phenotypes form physical borders in the TME.

Cancer related inflammation is characterized by a polarized distribution of macrophages at the site of the tumor. Macrophages are essential for promotion of cancer during both early and late stages of tumorigenesis. Pro-inflammatory M1 macrophages help counteract tumor growth by eliciting acute immune responses or direct killing *via* phagocytosis, while alternatively activated M2-like macrophages promote immune suppression, angiogenesis and tissue remodeling functions that sustain cancer growth in the sub-acute phase of the immune response (38). Within the TME, the presence of TAMs with a higher M2 to M1 ratio is linked to worse clinical outcomes, including poor survival rate, increased metastasis, and evasion of immune response (32, 42–46). Given these functional consequences of macrophage infiltration in cancer, and their critical role in iron metabolism in other organs of the body in the absence of malignancy, it stands to reason that tumor macrophages also are central to iron metabolism. Indeed, several studies have related immunological response and polarization with macrophage iron handling and cancer iron metabolism. The picture that emerges is one in which a macrophage “Iron Curtain” is established by the TME to directs iron flux and immune response towards tumor growth as shown in **Figure 2**. Here we review recent studies that have focused on the intersection of iron metabolism and macrophage polarization to generalize the cellular, and metabolic traits linking iron and immune response across cancers.

### Breast Cancer

Dysregulation of iron metabolism in breast cancer is evident by changes of protein gene expression and accompanies polarization of macrophages towards pro-tumor states. In a normal breast, unique populations of iron-handling M2 macrophages serve to regulate iron levels within the adipose-rich tissue to maintain normal adipogenesis and control peroxidative stress (24). In both murine and human breast tumor tissue, iron accumulation in TAMs is observed, with higher levels of TAM iron being associated with M1 polarization and less invasive cancer, while M2 polarization and reduced iron was observed in invasive breast cancer (47). Correlation of dysfunctional iron metabolism with breast malignancy is supported by differential expression of the high iron FE gene (HFE) gene variants in patients. Patients with major HFE variants have an increased risk of developing breast cancer (48). Iron associated proteins such as hepcidin, FPN, TfR1, and ferritin are highly expressed in breast tissue macrophages and lymphocytes in patients with the HFE variant gene, suggesting that increases in hepcidin and TfR1 favor tumor growth and lead to more aggressive forms of cancer (48). Findings by Pinnix et al. reveal a substantial reduction in FPN in breast cancer cells, where FPN abundance correlates with metabolically available iron. In this case, high levels of FPN and low hepcidin expression demonstrates a favorable cohort of breast cancer patients with an increased survival rate (49). Macrophages associated with breast cancer express high levels of ferritin light chain that promotes the M2 macrophage phenotype and fosters a pro-tumor environment in breast cancer by secreting ferritin iron into the stroma (43, 45). TAMs in more aggressive forms of breast cancer secrete lipocalin 2 (Lcn2), a small molecule that increases iron concentration and the iron labile pool of cancer cells within the TME to promote growth and resistance to chemotherapy (50, 51). The upregulation of Lcn2 significantly increases the iron concentration at metastatic tumor stages. while Targeting Lcn2 iron secretion for inhibition could starve cancer cells of iron, being a potential therapy to reduce tumor growth.

### Central Nervous System (CNS) Cancer

Microglia are the resident macrophages of the CNS that provide immune surveillance and play central roles in iron metabolism. Microglial polarization response to inflammation or wound healing cues accompanies shifts in iron regulatory proteins such as TfR, FPN, ferritin and others that signals the accumulation or release of iron, respectively (24). In brain cancers microglia and blood-derived macrophages have a variety of functional differences in tumor immune response, including iron metabolism. The genes that regulate iron uptake (CD163 and TfR1), metabolism, storage (ferritin light and heavy chain, NCOA4), and catabolism (HO-1) are more highly expressed in bone marrow derived macrophages, revealing that bone marrow derived macrophages sustain an iron-recycling metabolism. These bone marrow derived macrophages are known to infiltrate glioma and glioblastoma where their association with M2 immunosuppressive functions increases towards the middle of the tumor (44, 52). Pathological studies of brain tumors such as brain metastasis from breast cancer show that TAM populations associated with the growing tumor edge have higher iron levels than the center consistent with an M1 to M2 polarization gradient (53). Interestingly, recent studies of leptomeningeal metastasis have shown that similar to breast cancer cells, these cancer cells utilize Lcn2 to obtain iron from macrophages in the CNS space which promotes tumor growth (54). Future studies will shed light on whether this cooption of metabolic function occurring in the CSF is generalizable to other cancers and how macrophages mediate this iron transfer in accord with their polarization state.

### Lung Cancer

Alveolar macrophages have been recently implicated in iron trafficking and may exhibit some independence from the hepcidin/FPN axis (24), but macrophage polarization, as well as TfR, ferritin, and FPN expression within the lung predicts iron-recycling activity similar to other localized macrophages (55). Human lung adenocarcinoma and mouse models of Lewis lung carcinoma (LLC) with elevated levels of M2-like TAMs have poorer clinical outcomes, such as increased tumor growth, metastasis, and reduced survival compared with M1-like TAM infiltration (46). In lung adenocarcinoma and LLC, iron and heme can repolarize TAMs from the M2 to M1 cytotoxic phenotype, leading to direct tumor killing and reduced tumor growth. TAMs loaded with iron have low expression of FPN and are CD163, CD86, and HO-1 positive which are expected to prevent supplying iron to the tumor, thereby inhibiting growth. Patients with lung adenocarcinoma that accumulate iron show more M1 TAMs along with improved survival (42, 46). TAM exposure to heme or iron promotes an anti-cancer immune response by repolarizing TAMs to harness their direct tumor killing ability. Increasing the amount of iron loaded TAMs can be used as a potential therapy to help counteract tumor growth and increase patient survival.

### Kidney Cancer

In renal cell carcinoma (RCC), analysis of iron metabolism genes, FPN, ferritin light and heavy chains, IRP2, and TfR1, revealed that these genes are all highly expressed in RCC, similar to other cancers, and TfR1 expression is used as a biomarker of RCC and is associated with worse survival outcomes (56). Iron levels are elevated in RCC as well as genes responsible for iron handling, where this cancer depends on iron for escape of apoptosis and cell cycle arrest. The role of iron in kidney cancer was also linked to the von Hippel Lindau (VHL)/hypoxia inducible factor-α (HIF-α) axis, which is a major regulator of iron metabolism which is dysregulated in RCC. Iron dependency introduced by VHL inactivation reveals an interplay between VHL/HIF-α dysregulation and iron metabolism in RCC. TAMs were shown to have an M2 iron release phenotype with an increase of FPN receptor expression, promoting growth of RCC. To further confirm that iron promotes tumor growth, extracellular fluid from tumor tissue was applied to renal tumor cells, showing that proliferation along with metastasis was enhanced (32). These studies further identified that pathological iron accumulation occurs in TAMs compared with normal iron levels in kidney macrophages. It is intriguing to suppose that TAMs in kidney cancer contribute to tumor proliferation and dissemination by sustaining an iron release phenotype *via* upregulation of FPN and M2 polarization.

### Prostate Cancer

Metabolic iron feedback between prostate cancer cells and macrophages provides a putative connection between macrophage infiltration and tumor iron dysfunction observed in prostate cancer. Prostate cancer cells are highly dependent on iron for their proliferation (57). In this iron addicted state, they exhibit a low FPN high TfR phenotype and synthesize hepcidin to induce neighboring tissue iron retention to support their cellular program. Approximately 80% of prostate cancer patients exhibit anemia of chronic disease (ACI), that paradoxically, is associated with iron accumulation in macrophages occurring *via* hepcidin signaling (58). While studies thus far have not definitively linked hepcidin signaling with macrophage polarization in prostate cancer, supporting the idea that prostate cancer cells induce non-heme macrophage iron in tumors clinical studies have found elevated non-heme ferritin tissue iron is associated with malignant tumors compared with benign (59, 60). Iron loading specifically in macrophages has also been observed in prostate cancer. Studies in mouse models show accumulation of macrophage iron in tumors and in systemic iron handling macrophage populations is related to tumor growth and extent of macrophage infiltration in the tumors (61). Generalizing these combined findings prostate cancer TAMs at the invasive margin of the tumor are associated with M1 polarization and high iron levels, where more invasive TAM found deeper in the tumor were primarily M2 polarized and have less iron stores.

### Hematological Malignancy

In addition to infiltrating macrophages of solid tumors, it is of interest to consider changes in iron metabolism that are brought about by malignancy involving myeloid cells themselves. Myelodysplastic syndromes constitute a diverse group of hematopoietic stem cell disorders resulting from ineffective hematopoiesis that can lead to acute myeloid leukemia (AML) (62). AML is a malignant hematologic disorder within the bone marrow, blood, and other tissues containing cells of the hematopoietic system (63). Cytotoxic chemotherapy and ineffective hematopoiesis contribute to iron accumulation in these patients and serum ferritin levels have been correlated with an increased risk of relapse (64). Clinical studies have shown that elevated levels of serum ferritin were associated with poor prognosis in patients with hematological malignancies (65). However, serum ferritin concentration is controversial in determining a prognosis in patients with AML because chronic blood transfusion commonly seen in patients with myelodysplastic syndromes and AML that improve anemia and increase the quality of life, also exacerbates iron loading confounding the prognostic value of serum ferritin (63).

## Immune Response and Iron Metabolism in Cancer Therapy

**Figure 3:**
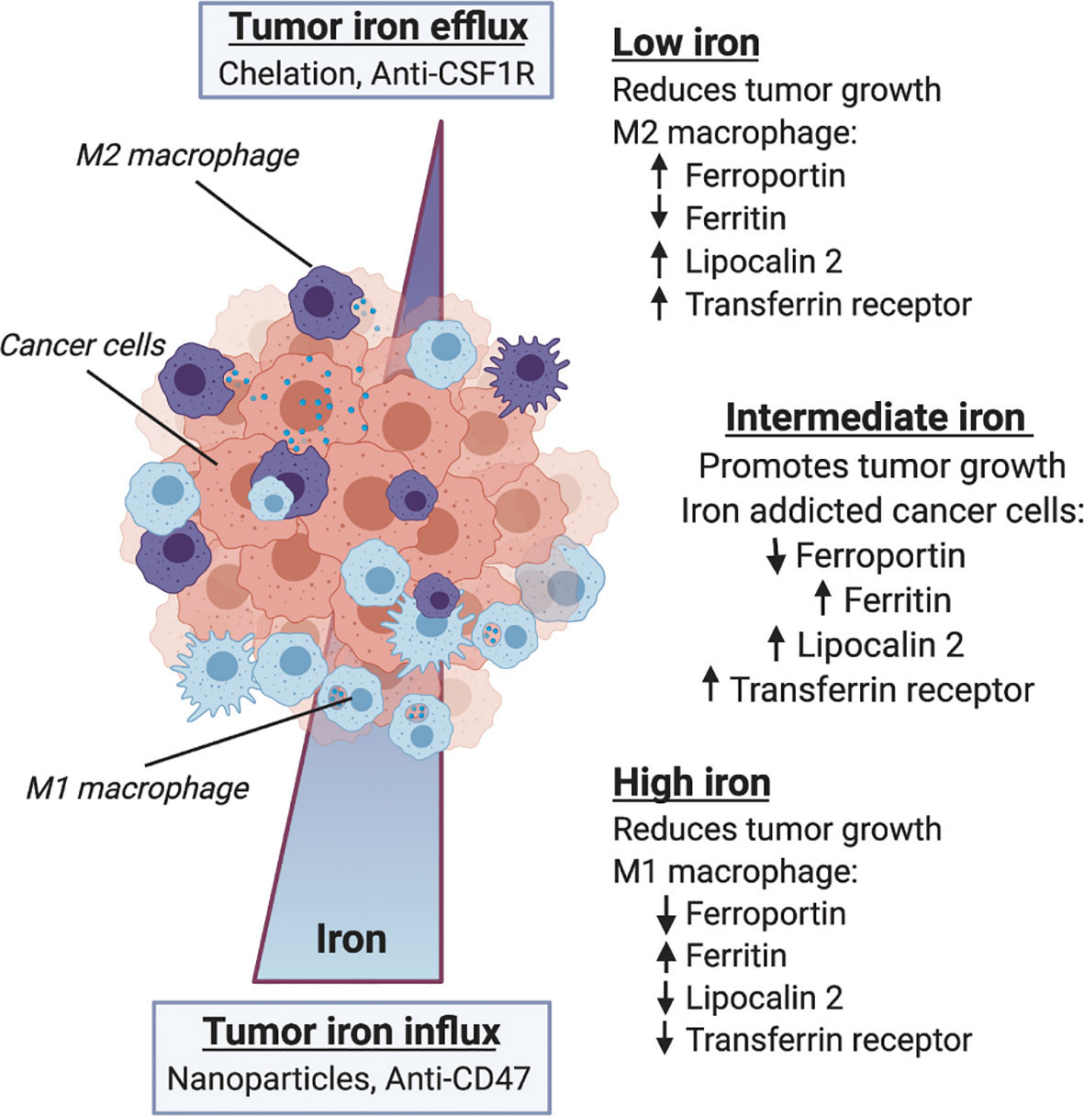
Macrophage iron metabolism and immune response in the tumor microenvironment. Within the microenvironment macrophage phenotype is influenced by iron metabolism, and both iron and immune status are correlated with tumor growth and therapy response. Along a gradient of tumor iron concentration established according to systemic metabolic background and mode of therapy, macrophages can adopt various polarization states spanning a continuum between M1 anti-tumor/proinflammatory activation and M2 pro-tumor wound-healing states. Low tumor iron is associated with reduced tumor growth and favors M2-like macrophage polarization with increased expression of ferroportin, lipocalin 2 and transferrin receptor, and reduced ferritin content. High tumor iron is similarly associated with reduced tumor growth and favors M1-like macrophage polarization with lower expression of ferroportin, lipocalin 2 and transferrin receptor, and increases in ferritin iron storage. Between these two extremes the heterogeneous distribution of macrophage polarization states supports an intermediate iron regime where iron-addicted cancer cells expressing low levels of ferroportin, and high levels of ferritin, lipocalin 2 and transferrin receptor co-opt macrophage’s innate role in iron handling to support malignancy.

The above studies provide evidence supporting the central role of iron in tumor growth that is regulated in the microenvironment by macrophages. However, it remains unknown whether changes in iron metabolism stimulate changes in immune status and response, or whether changes in immune status effect macrophage iron handling. Further, it is still unknown whether these metabolic and immunologic responses arise from microenvironmental cues, or if systemic changes in iron metabolism and immune response dictate the iron handling and immune response of TAMs locally. We can derive some insight into these mechanisms by examining therapeutics targeting either iron metabolism or immunity and evaluate their reciprocal effects. Tumor response to drugs such as iron-depleting chelators or iron accumulating nanoparticles can be evaluated to investigate the role of macrophages in transmitting systemic metabolic cues to the tumor-immune microenvironment. Similarly, tumor metabolism modulates immunotherapy response, and given the previously mentioned disruptions in immune cell signaling likely has effects on macrophage iron metabolism both systemically and in the tumor microenvironment such as shown schematically in **Figure 3**. Here we review several therapy studies that highlight the reciprocity between iron metabolism and immune response and place macrophages at the intersection of immune-metabolic processes as they relate to cancer therapy efficacy.

### Tumor Immune Response in Iron Chelation Therapy

Iron chelators, such as clinically used deferoxamine (DFO), deferiprone (DFP) and investigational chelators such as EC1 tropolone, have been shown to inhibit cancer cell growth *via* a variety of mechanisms, involving inhibition of iron-dependent processes *via* their role as enzymatic co-factors, catalysts for reactive species generation, and others (66, 67). Here we review several iron-chelation therapy studies in which immune response of macrophages is induced.

Iron chelators have been shown to alter iron metabolism, macrophage polarization, and immune signaling. Supporting these effects in cancer, DFO administration decreases iron availability from gastric cancer tissue slice cultures, reduces viability of cancer cells, and leads to high iron efflux by decreasing ferritin expression in the TME and TAMs (43). DFO also has effects on immune signaling cytokine factors in cancer. To characterize the relationship between TNF-α and iron metabolism during inflammation, the regulatory interactions between metabolism, cellular differentiation, and TNF-α release was investigated in the human monocyte cell line THP-1 with DFO (68). DFO decreased TNF-α expression and when added to phorbol‐12‐myristate‐13‐acetate (PMA) stimulated cells DFO rapidly inhibited TNF-α release. Addition of iron salts to PMA-differentiating cells increased TNF-α mRNA expression and protein release, supporting that iron may mediate the pro-inflammatory response. In other studies of DFO, the role of iron and ferritin have been investigated according to their role modulating MHC-I expression and natural killer cell signaling. Macrophages, critical innate immune cells and iron regulators, express MHC-I and II proteins to present antigens to other lymphocytes such as natural killer cells. When given the iron chelator DFO, MHC-I cell surface expression decreases together with degradation of ferritin and ferritin heavy chain shRNA (69). Additionally, in mouse models of ferritin accumulation, expression of MHC-I cell surface receptors was increased, and DFO reduced ferritin levels and MHC-I. This supports a role for iron chelators in ferritin regulation of iron metabolism in parallel to their inhibitory effects on inflammatory immune signaling factors such as TNF-α and MHC-I.

DFP is another FDA approved small molecule iron chelator prescribed in cases of iron overload. The mechanism of action of deferiprone is similar to DFO, but the two agents different in that DFO chelates extracellular iron while DFP is an intracellular iron chelator. This allows DFP to mobilize cellular iron which is likely related to its effects on cancer cell proliferation. The efficacy of DFP has also been evaluated in the context of macrophage iron accumulation. Macrophages sequester iron as hemosiderin, ferritin protein aggregates, and are known as hemosiderin laden macrophages (HLM) to prevent depletion of iron and maintain levels of cytotoxic free iron. This ability of macrophages to store and metabolize iron puts them in a position to be used as putative iron reservoirs that can be exploited by tumor cells to promote their growth. In vivo treatment of Myc-CaP and TRAMP-C2 mice models of prostate cancer with DFP led to a significant anti-tumor response that was directly proportional to the amount of iron found in tumor, tumor associated macrophages and peripheral macrophages of the liver and spleen as detected by immunopathology and MRI (61). Importantly, these studies enabled the direct *in vivo* observation of the “Iron curtain” which defined a new prognostic biomarker of macrophage iron handling associated with their spatial infiltration and cancer therapy response.

Supporting the role of macrophages in providing iron to support tumor growth, the iron chelator, EC1 was investigated. EC1 is a thiosemicarbazone chelator with a tridentate binding unit that ensures high affinity iron binding. The role of macrophage secreted iron was examined in renal cell carcinoma cell lines and patient samples of tumor progression by applying this novel chelation approach (32). These authors found that iron regulating genes were significantly upregulated in tumors when compared to healthy tissue with tumor cells retaining iron and TAMs exhibited an iron releasing M2-like phenotype. Iron concentration increased in macrophage extracellular fluids which when added to tumors stimulated tumor growth. Macrophage derived iron had pro-tumor functions but was seen to be blocked once EC1 chelator was administered. The addition of EC1 reversed the effect of macrophage conditioned media on cancer cell proliferation and reduced the effect of iron supplementation on tumor cell proliferation and migration. This study shows that the labile iron pool in the TME is regulated by tumor macrophages which drives cancer, and that this interaction can be disrupted by small molecule iron chelators to reduce tumor growth.

### Tumor Immune Response to Iron

The above studies highlight the ability of systemically administered iron chelators to interfere with the iron-regulating functions of macrophages, which has complementary effects on microenvironmental immune response and tumor growth. As counterpoint to these studies, we can consider effects of iron accumulation rather than depletion *via* chelation and evaluate how macrophages handle increases in systemic iron concentrations in the tumor microenvironment.

Given the association of iron accumulation with M1-type macrophage function, many investigators have proposed the hypothesis that increasing iron in macrophages as cancer therapy can induce this effect and thereby stimulate anti-tumor immune response, including cytokine formation. Short-term iron overload has been associated with production of TNF-α and long-term iron overload leads to inactivation of macrophages, reflected by decreased TNF-α, IL-6, IL-12, MHC-II, ICAM1, and iNOS expression (70, 71). This leads to induction of anti-inflammatory pathways and impaired control in numerous infectious diseases (72). This also has an impact on the effects of iron chelators, which have been shown to promote the M1-like macrophage phenotype (73, 74). Iron loading may also result in de-activation of macrophages *via* induction of HO-1, resulting in tolerance developing, where the presence of tumor cells is tolerated (75).

**Figure 4:**
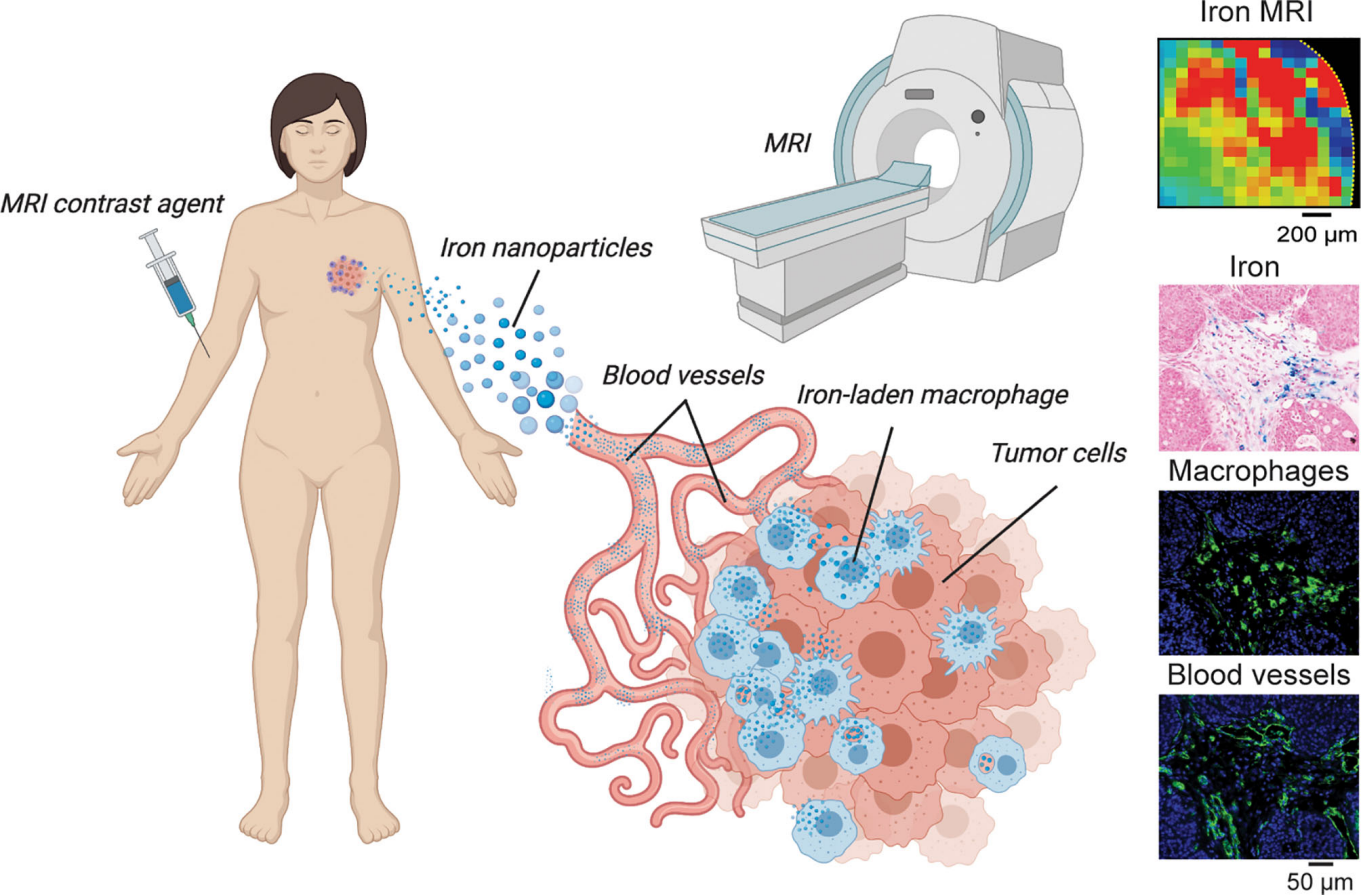
TAM iron imaging in cancer. In many studies focusing on iron-laden population of macrophages magnetic resonance imaging (MRI) is used to localize and monitor these cells during tumor growth and immunometabolic therapy response. Here, iron nanoparticle contrast agents are injected intravenously and subsequently are delivered to the tumor where TAM phagocytosis occurs. Quantitative iron MRI provides *in vivo* quantitative detection of iron containing macrophages in animal models and patients. Cytological imaging confirms associations between iron deposits within macrophage accumulation and vascular infiltration.

Iron nanoparticle injection has been shown to induce M1 polarization, triggering apoptosis of cancer cells *via* an autocrine feedback loop that maintains TNF-α and nitric oxide within the TME to continuously inhibit tumor growth, reduce cell migration, and inhibit pulmonary and hepatic metastasis (76). These nanoparticles are also commonly used as TAM imaging contrast agents in MRI preclinically and clinically as shown in **Figure 4**. Such nanoparticles are able to polarize RAW264.7 macrophages to an M1-like phenotype characterized by elevated expression levels of TNF-α, INOS, CD11b, and CD80. ROS is also enhanced in tumor cells by iron nanoparticles that triggers caspase 9 expression and apoptosis (77). Additionally, nanoparticles alone and in combination with other therapeutic intervention such as photothermal therapy promote tumor associated antigen release and recruitment of T-helper and T-effector cells at the tumor site through repolarization of M2 TAMs to the M1 phenotype (78). This indicates that due to the dependence on both cancer cells and macrophages on iron, these nanoparticles have pleiotropic effects on immune response in tumors that can be exploited to induce transient anti-tumor responses involving oxidative stress.

### Iron Metabolism and Macrophage-Targeted Immunotherapy

In the same light that we consider iron chelation as promoting iron release and depletion from the tumor, here we begin with discussion of immunotherapies that are reported to have similar cellular iron reducing effects. The colony stimulating factor 1 receptor (CSF1R) is a key regulator of monocyte function that drives the recruitment of macrophages to the TME and promotes their differentiation to pro-tumorigenic TAMs (79–81). Preclinically, inhibition of CSF1R using monoclonal antibodies (mAbs) or small molecule drugs, such as BLZ945 and PLX3397, have been used to treat malignancies including breast, ovarian, brain, pancreatic and other cancers where they decrease TAM accumulation and promote tumor growth inhibition (47, 82–84). The overexpression of CSF1R has been associated with poor prognosis in many cancers and accumulated evidence has made it clear that combination of CSF1R immunotherapy with other standard-of-care often improves therapeutic response which is currently of clinical interest (85, 86).

On the other side of the coin, we can also consider potential effects of immunotherapies on inducing cellular iron accumulation in the tumor. For example, CD47 expression is an independent poor prognostic marker and serves as the ligand for signal-regulatory protein alpha (SIRPα), present on macrophages and other phagocytic cells that inhibits phagocytosis when activated. Monoclonal antibodies against CD47 (CD47 mAb) inhibit the interaction between SIRPα and CD47 effectively blocking the “don’t eat me” signal to activate TAMs and promote macrophage phagocytosis of, for example, of malignant osteosarcoma cells (87), and self-renewing leukemia stem cells (LSC) that promote AML (88). To prevent tumor dissemination, CD47 mAb may be administered systemically or locally upon surgical resection to eliminate circulating tumor cells (89). Given the role of macrophages in recycling iron *via* cellular phagocytosis, anti-CD47 immunotherapy likely effects tumor iron metabolism. This has been investigated by Daldrup-Link and coworkers where combination of doxorubicin and CD47 mAb significantly inhibited tumor growth and improved survival in a manner proportional to the increase in iron metabolism that was detected by increases in TAM iron using histology and MRI (90). Outside of the tumor microenvironment, one notable effect on iron metabolism that occurs with anti-CD47 therapy is the onset of systemic anemia which likely has contributions from over-accumulation of iron in tissue macrophages that can lead to reductions of the systemic availability of iron for RBC heme synthesis (91–93). This effect would suggest that anti-CSF1R also effects systemic iron metabolism whereby reduced macrophage iron accumulation vis a vis cellular macrophage depletion would increase peripheral iron availability, but this mechanism has yet to be proven.

### Immune Checkpoint Blockade and Iron Metabolism

The ligand for programmed cell death protein 1 (PD-L1) is frequently overexpressed on tumor cells enabling their escape from immune surveillance. Monoclonal antibodies blocking PD-L1/PD-1, so called immune checkpoint blockade inhibitors (ICB), have been clinically shown to have efficacy in patients with a variety of cancers by activating T lymphocytes (94, 95). While iron metabolism *per se* has not been an area of focus in adaptive immunotherapy to date, research on T lymphocyte biology have noted that iron metabolism plays an important role in T cell migration and activation throughout the body (25, 96, 97). Specific involvement of macrophage iron metabolism in instances of ICB nonetheless can be speculated from recent studies. For example, along with T cells, PD-1 blockade rescues macrophage and dendritic cell function in the TME, activating the immune cells against the tumor. It was shown that TAM PD-1 expression is negatively correlated with phagocytotic potency against tumor cells, but blockage of PD-1/PD-L1 *in vivo* increased macrophage phagocytosis, reduced tumor growth, and increased survival (98). Considering the effects that anti-CD47 has on macrophage iron accumulation, these similar observations during ICB suggest a corresponding effect on macrophage iron accumulation. Additionally, anemia has been reported as a correctable but significant effect in a clinical ICB trials that further suggests a parallel between systemic iron metabolism in ICB and the metabolic status observed during direct targeting of macrophage by other immunotherapies (99, 100).

An area of additional relevance relating effect of ICB with iron metabolism comes from new developments in the field of cell death, specifically in the context of ferroptosis. Ferroptosis is a novel cell death mechanism that proceeds *via* iron catalyzed peroxidation of polyunsaturated lipids and its’ regulation by factor, such as system Xc^-^ and the glutathione peroxidase 4 enzyme (GPX4) which maintains cellular redox homeostasis (101). Sensitivity of cancer to this cell death mechanism is attributed to a variety of factors such as tissue iron, lipid composition, and the expression of redox regulating proteins (102–104). Thus, macrophages likely represent a central cellular player in the mechanism as they mediate iron storage and release in the tissue microenvironment. Indeed, the iron accumulating properties of M1 macrophages have been implicated in driving ferroptosis by harboring higher concentrations of iron that under appropriate conditions brought about by iron challenge or ferroptosis-targeted agents that block reactive species scavengers, can increase levels of lipid peroxides and sustain ferroptotic cell death (105–108). In ICB cancer immunotherapy, tumor growth inhibition caused by the drug is associated with increased lipid peroxidation and can be further amplified by ferroptosis-targeted drugs and significantly inhibited by iron chelators (109). While this suggests that modulation of iron metabolism is a clear avenue for altering immunotherapy efficacy, the field of ferroptosis must still address some outstanding questions regarding tolerance of ferroptosis-targeted agents, and more fundamentally, how to reconcile the association of increased iron and lipid peroxidation in driving ferroptosis with the same associations of these metabolic factors in also increasing oxidative stress and causing peroxidative DNA damage that promotes carcinogenesis (76, 110, 111). The balance between ferroptotic cell death and peroxidative carcinogenesis in the context of tumor susceptibility to iron chelators is of special significance regarding efficacy of susceptibility to ferroptosis-targeted drugs and their combinations with ICB in the context of iron metabolism and immune response. These observations support further investigation into macrophage iron handling as a critical factor in ICB response that can potentially be harnessed by tapping into ferroptosis pathways to improve this mode of cancer therapy.

### Iron Metabolism and Adoptive Cell Therapy

Adoptive cell therapy seeks to modulate the immune response by engineering immune cells from the patient and reintroducing them to reset the patient’s immune system (112). Currently, the most pursued adoptive cell therapy involves chimeric antigen receptor (CAR) T cells that are engineered to express receptor binding motifs tethered to T cell activating constructs that when re-introduced to a patient bind and eliminate specific malignancies such as leukemias and lymphomas, and increasingly, solid tumor cancers. One major drawback of these therapies has been patient toxicity due to the rapid and amplified immune response these agents induce. This so-called cytokine release syndrome, or storm, that results from these infusions and their target interactions is part of the acute phase response (113). One of the signatures of the acute phase response linking adoptive cell therapy to iron metabolism is elevated serum ferritin (114, 115). Definitive connections between this metabolic iron response in cytokine storm and macrophages have not been drawn in CAR-T cell therapy. However, investigators have shown that macrophages are central mediators of the cytokine storm and that inhibiting their signaling, for example by targeting IL-6 or granulocyte-macrophage colony stimulating factor (GM-CSF), can alleviate these over-responses while maintaining therapeutic efficacy (116, 117). Further, iron homeostasis can affect migration, function, and differentiation of T lymphocytes, therefore not only does iron metabolism effect TAMs, but also T cells and other tumor-infiltrating lymphocytes are directly and/or indirectly affected. Iron has been shown to trigger CD4+ T cell differentiation and alter CD8+ T cell expansion. Immunosuppressive effects of iron on T cells have been described in individuals with hereditary or transfusion mediated iron overload, where these patients have altered T cell numbers and function (118). In tumor infiltrating lymphocytes, iron may impair the proliferation, differentiation or maturation by generating mitochondrial ROS, resulting in cell death. Pursuit of this role of macrophages and lymphocytes in mediating ferritin iron efflux is likely to be an important correlate of adoptive cell therapy and ensuing cellular responses. 

## Concluding Remarks

Macrophage infiltration in cancer is associated with poor patient outcomes and therapy resistance. There is an incomplete understanding of the balance between macrophage polarization and functional phenotype related to these effects. Dysfunctional primary metabolism is a hallmark of cancer cells that is now accepted as contributing to the pro- versus anti-tumor response decisions of macrophages. As we review above, it is evident that iron metabolism plays a major role in the cellular plasticity of tumor macrophages and their involvement in cancer. Here we considered two aspects of macrophage function to advance our understanding of this interaction space. The first is the role of macrophages as central regulators of systemic iron metabolism. The second is their contribution to the immune landscape of the tumor microenvironment. At the intersection of these two critical roles, we focused on a metabolically distinct cellular population of iron-containing macrophages. We find that unique populations of macrophages throughout the body and in tumors perform similar iron handling functions where macrophage iron accumulation and release in the tissue is synchronized with the phase of systemic and microenvironmental immune responses. In tumors specifically, sites of inflammation are indicated by M1 polarized tumor-associated macrophages (TAM) which accumulate iron, accumulation while release of iron to cancer cells by M2 polarized macrophages supports the iron-addicted metabolic program of the cancer cells within the TME.

To fully establish the role of macrophage iron metabolism in cancer, however, more research is needed to answer pressing questions such as: How spatial heterogeneity in tumor microenvironment influences the molecular networks that allow iron exchange between cancer cells and macrophages? How iron flux in macrophages changes their communication with other immune cell and stromal components? How systemic metabolic dynamics of iron alters macrophage plasticity longitudinally over the many complex steps of tumorigenesis, cancer progression, metastasis, and therapeutic response? In the future we will tackle these multiscale phenomena from a bench-to-beside approach, making use of translational advances in cytology, bioinformatics, and imaging to reveal new ways to therapeutically harness these targets and translate these insights to the clinic. Our insights here support these further investigations into the crosstalk between iron metabolism and immune response and strongly warrants further development of anti-cancer therapies that target this immunometabolic axis.

## Author Contributions

AD and AL conducted literature searches, prepared figures, and wrote the article. All authors contributed to the article and approved the submitted version.

## Funding

We gratefully acknowledge funding from the Carol M. Baldwin Breast Cancer Research Fund, Stony Brook School of Medicine Departments of Radiology and Pharmacological Sciences, and the Bahl Center for Metabolomics and Imaging at the Stony Brook Cancer Center.

## Conflict of Interest

The authors declare that the research was conducted in the absence of any commercial or financial relationships that could be construed as a potential conflict of interest.
